# Environmental Enrichment Rescues Endocannabinoid-Dependent Synaptic Plasticity Lost in Young Adult Male Mice after Ethanol Exposure during Adolescence

**DOI:** 10.3390/biomedicines9070825

**Published:** 2021-07-16

**Authors:** Irantzu Rico-Barrio, Sara Peñasco, Leire Lekunberri, Maitane Serrano, Jon Egaña-Huguet, Amaia Mimenza, Edgar Soria-Gomez, Almudena Ramos, Ianire Buceta, Inmaculada Gerrikagoitia, Juan Mendizabal-Zubiaga, Izaskun Elezgarai, Nagore Puente, Pedro Grandes

**Affiliations:** 1 Department of Neurosciences, Faculty of Medicine and Nursing, University of the Basque Country UPV/EHU, 48940 Leioa, Spain; irantzu.rico@ehu.eus (I.R.-B.); leire.lecumberri@ehu.eus (L.L.); maitane.serrano@ehu.eus (M.S.); jon.egana@ehu.eus (J.E.-H.); amaia.mimenza@ehu.eus (A.M.); edgar.soria@achucarro.org (E.S.-G.); almudena.ramos@ehu.eus (A.R.); ianire.buceta@ehu.eus (I.B.); inma.gerrikagoitia@ehu.eus (I.G.); juanluis.mendizabal@ehu.eus (J.M.-Z.); izaskun.elezgarai@ehu.eus (I.E.); nagore.puente@ehu.eus (N.P.); 2 Achucarro Basque Center for Neuroscience, Science Park of the UPV/EHU, 48940 Leioa, Spain; 3 Instituto de Investigación Biomédica de Málaga (IBIMA), Hospital Regional Universitario de Málaga, 29010 Málaga, Spain; sara.penasco@ibima.eu; 4 IKERBASQUE, Basque Foundation for Science, 48013 Bilbao, Spain; 5 Division of Medical Sciences, University of Victoria, Victoria, BC V8P 5C2, Canada

**Keywords:** endocannabinoid system, synaptic plasticity, memory, drug addiction, enrichment therapy

## Abstract

Binge drinking (BD) is a serious health concern in adolescents as high ethanol (EtOH) consumption can have cognitive sequelae later in life. Remarkably, an enriched environment (EE) in adulthood significantly recovers memory in mice after adolescent BD, and the endocannabinoid, 2-arachydonoyl-glycerol (2-AG), rescues synaptic plasticity and memory impaired in adult rodents upon adolescent EtOH intake. However, the mechanisms by which EE improves memory are unknown. We investigated this in adolescent male C57BL/6J mice exposed to a drinking in the dark (DID) procedure four days per week for a duration of 4 weeks. After DID, the mice were nurtured under an EE for 2 weeks and were subjected to the Barnes Maze Test performed the last 5 days of withdrawal. The EE rescued memory and restored the EtOH-disrupted endocannabinoid (eCB)-dependent excitatory long-term depression at the dentate medial perforant path synapses (MPP-LTD). This recovery was dependent on both the cannabinoid CB1 receptor and group I metabotropic glutamate receptors (mGluRs) and required 2-AG. Also, the EE had a positive effect on mice exposed to water through the transient receptor potential vanilloid 1 (TRPV1) and anandamide (AEA)-dependent MPP long-term potentiation (MPP-LTP). Taken together, EE positively impacts different forms of excitatory synaptic plasticity in water- and EtOH-exposed brains.

## 1. Introduction

Abusive EtOH consumption typically initiates during adolescence [1], having severe toxic effects on the maturing brain that leads to long-lasting neuropsychological alterations [2]. Recent studies have demonstrated that adolescent BD alters neurotransmitter systems [3], reduces neurogenesis [4], and affects motor coordination and balance [5]. In addition, EtOH intake causes changes in glial cells [6,7], activates immune responses [8], and induces a significant cognitive impairment through the modulation of synaptic transmission and plasticity, notably in the hippocampus [9].

The endocannabinoid system (ECS) is formed by the G-protein coupled cannabinoid CB1 and CB2 receptors (CB1R, CB2R), the main endocannabinoids (eCBs) 2-arachydonoyl-glycerol (2-AG) and anandamide (AEA), as well as the enzymes involved in their synthesis, degradation, and transport [10,11]. This system plays important roles in synaptic plasticity and interacts with EtOH in a reciprocal manner. Chronic EtOH alters CB1R expression, density, and functionality [12,13,14,15,16]. EtOH suppresses CB1R depression of field excitatory postsynaptic potentials (fEPSPs) evoked by medial perforant path (MPP) stimulation in the dentate gyrus molecular layer (DGML) of the hippocampus [17]. EtOH intake during adolescence also negatively impacts the CB1R-dependent eCB-eLTD that is mediated by mGluR1 and mGluR5, intracellular calcium, and 2-AG synthesis at the MPP-granule cell (MPP-GC) synapses under normal conditions [17,18]. Moreover, the reduced CB1R functionality in the hippocampus, the decrease in excitatory terminals in the DGML, and the rise in monoacylglycerol lipase (MAGL) detected after adolescent EtOH intake [17] associate with long-lasting memory impairment [5,19].

An enriched environment (EE) provides multisensory stimulation [20,21,22] that increases neurotrophic factors [23,24], adult hippocampal neurogenesis [25], and structural plasticity [26,27]. Consequently, an EE favors cognitive functions in both physiological and pathological conditions [28,29,30], including drug addiction [31]. However, despite EE recovering long-lasting motor coordination, balance, and cognitive impairment after BD during adolescence [5], little is known about the underlying mechanisms. In this study, we investigated the EE effects on the altered CB1R-dependent synaptic transmission and MPP-LTD that occur in the young adult mouse hippocampus after chronic EtOH intake during adolescence. The results reveal that an EE recovers memory through the rescue of the MPP-LTD. An EE on water-exposed conditions results in TRPV1 and AEA-mediated MPP-LTP. These findings suggest the potential benefits of EE in the normal (water-exposed) and EtOH-exposed brain through different cannabinoid-dependent synaptic plasticity mechanisms.

## 2. Materials and Methods

### 2.1. Experimental Animals and Ethics Statement

Three-week-old C57BL/6J male mice (Janvier Labs, Le Genest-Saint-Isle, France) were randomly distributed in pairs of control and EtOH experimental groups. Animals were maintained at 22 °C in a temperature-controlled room with a 12 h light and dark cycle (red light on at 9:00 a.m.) to habituate to the new environment 1 week before experimental procedures were initiated. All procedures were approved by the Committee of Ethics for Animal Welfare of the University of the Basque Country (CEEA/M20/2016/073; CEIAB/2016/074) and were in accordance with the European Communities Council directive of 22 September 2010 (2010/63/EU) and Spanish regulations (Real Decreto 53/2013, BOE 08-02-2013). Ad libitum access to food and water was available along all experiments except during DID procedure.

### 2.2. DID Procedure and Timeline

**Figure 1:**
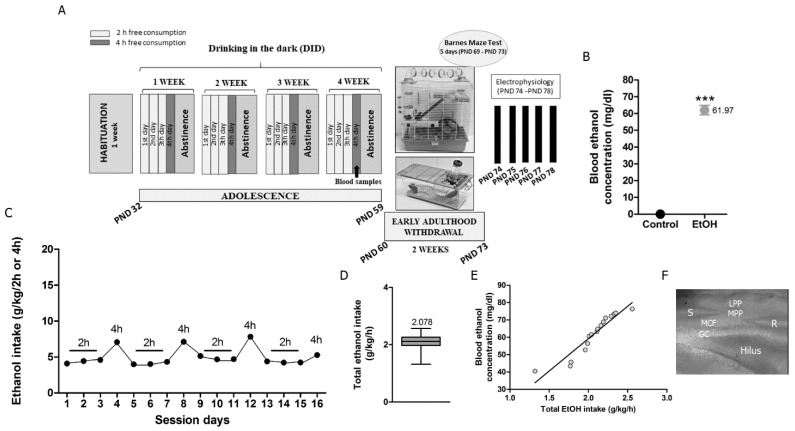
Schematic representation of experimental timeline, BEC, daily voluntary oral EtOH intake, total EtOH intake, and correlation between total EtOH intake and BEC. (**A**) Male C57BL/6J mice were exposed to 4 weeks DID during adolescence (PND 32–56). Each week, mice were exposed to 2 and 4 h of free EtOH access (20% (*v*/*v*)). Several mice were under environmental enrichment over the 2 weeks of withdrawal (PND 60–73) and were subjected to the Barnes maze test in the last 5 days (PND 69–73). Then, they were sacrificed to perform physiological recordings at early adulthood (PND 74–78). (**B**) BEC obtained on the last day of EtOH exposure averaged 61.97 ± 2.84 mg/dL ((*n* = 16) Student’s *t*-test; *** *p* = 0.0001 vs. control). (**C**) EtOH intake during each day (g EtOH/kg/2 or 4 h) was measured and (**D**) total EtOH intake averaged 2.078 ± 0.07 g/kg/h (*n* = 16), similar to previous studies [5,17,32]. (**E**) A direct correlation between total EtOH intake and BEC was detected. (**F**) Stimulating electrode (S) was placed in the middle one-third of the DGML to stimulate MPP. R recording electrode, GC granule cells, MCF mossy cell fibers, MPP medial perforant path, LPP lateral perforant path.

Four-week-old adolescent male mice [postnatal day (PND) 32] were classified in either control or EtOH experimental groups. Mice were housed in pairs in standard 17 × 14.3 × 36.3 cm plexiglas cages. They were exposed to 4-day DID [32] over 4 weeks (PND 32–56). On days 1–4 of each week, mice were weighed at 8:00 a.m. 1 h before lights off. At noon (3 h into the dark cycle), they were individually placed into a cage provided with a single bottle of 10 mL tap water or a single bottle of 10 mL EtOH [20% EtOH (*v*/*v*) prepared from 96% EtOH (Alcoholes Aroca S.L., Madrid, Spain)]. Mice had free access to water (control group) or EtOH (EtOH group) for 2 h the first 3 days, and for 4 h the fourth day. They were kept resting in abstinence the last 3 days of each week with access to food and tap water ad libitum (Figure 1A). Thirty minutes after the last 4 h exposure, blood samples were collected from the lateral tail vein, and the blood ethanol concentration (BEC) (mg/dL) was measured in each mouse using a commercial EtOH assay kit (Sigma-Aldrich, Madrid, Spain) (Figure 1B). The effectiveness of the DID procedure was demonstrated by measuring EtOH intake during each day (grams of EtOH per kilogram per 2 or 4 h (g/kg/2 or 4 h)) (Figure 1C) and total EtOH intake (grams of EtOH per kilogram per hour (g/kg/h)) (Figure 1D). The correlation between total EtOH intake (g/kg/h) throughout DID and BEC (mg/dL) measured at the end of EtOH access was also calculated (Figure 1E).

### 2.3. Enriched Environment

Mice were in withdrawal for 2 weeks after DID (PND 60–73). Half litters of EtOH-exposed and control mice were placed into EE conditions (groups EE-EtOH and EE-H_2_0), while the others remained in standard conditions (groups EtOH and control) (Figure 1A). The EE consisted of a large two-level cage (50 cm length × 28 cm height × 54 cm width) equipped with nesting material, a little house, climbing ladders, a running wheel, tunnels, and toys of different colors, sizes, and material (plastic, wood, and metal). The toys were changed and rearranged twice a week in order to increase exploratory capacity and maintain novelty. Seven mice per cage were housed to ensure social interactions with food and water provided ad libitum.

### 2.4. Barnes Maze Test

**Figure 2:**
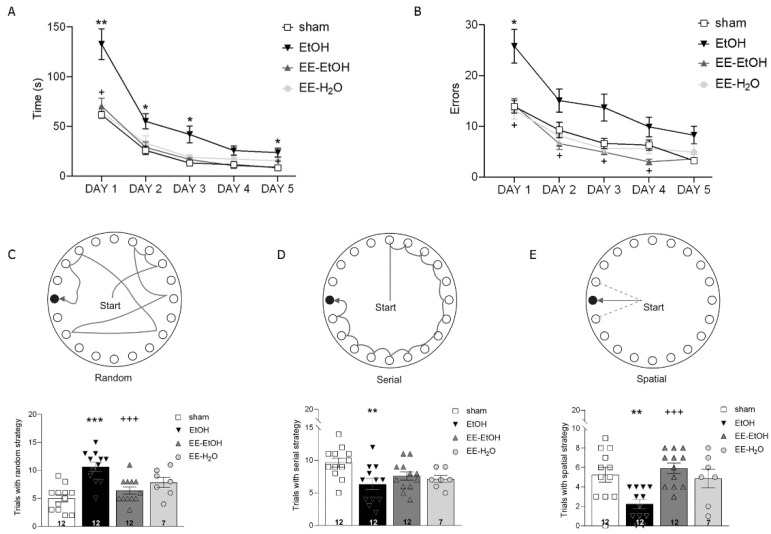
Barnes maze performance; EE recovers EtOH-induced memory impairment. (**A**) Latency to enter the escape box; time of each training day. (**B**) Holes visited before finding the escape box (errors); mean errors per day. (**C**) Random, (**D**) serial, or (**E**) spatial strategy chosen by each group (sham, EtOH, EE-EtOH and EE-H_2_O). Data are expressed as mean ± SEM. *n* = 12. * significance sham vs. EtOH, EE-EtOH, and EE-H_2_O (** *p* ≤ 0.01; *** *p* ≤ 0.001). ^+^ significance EtOH vs. EE-EtOH and EE-H_2_O (^+++^ *p* ≤ 0.001). Each symbol represents one mouse.

Learning and memory were tested using the Barnes maze [33]. The escape hole randomly selected for the mouse was maintained for each daily trial. The escape box under the selected hole was not visible; therefore, the mice had to use different cues placed in the room to discover it. The task consisted of 4 trials/day over 5 days. In each trial, the mouse was allowed to explore for a maximum of 240 s or until the escape box was found. If not found, the experimenter gently guided the animal into the box. The mouse rested in the box for 60 s after the trial. They were able to use different strategies to find the escape box (Figure 2C–E): random, serial, and spatial. The latency to escape, errors (non-target holes visited before escaping), and the strategy used to escape were quantified.

### 2.5. Slice Preparation and Extracellular Field Recordings

Young adult C57BL/6J male mice (*n* = 3 for each experimental condition, PND 74–78) were anesthetized by inhalation of isoflurane (2–4%). After decapitation, brains were carefully removed and submerged in a sucrose-based solution, measured in mM: 87 NaCl, 75 sucrose, 25 glucose, 7 MgCl_2_, 2.5 KCl, 0.5 CaCl_2_, and 1.25 NaH2PO_4_. Then, 300 μm-thick coronal hippocampal slices were cut using a vibratome (Leica Microsistemas S.L.U., Barcelona, Spain). They were collected into a recording chamber containing artificial cerebrospinal fluid (aCSF) comprised of (in mM): 130 NaCl, 11 glucose, 1.2 MgCl_2_, 2.5 KCl, 2.4 CaCl_2_, 1.2 NaH2PO_4_, and 23 NaHCO_3_. And maintained at 32–35 °C while continuously bubbled with 95% O_2_/5% CO_2_. Picrotoxin (Tocris BioScience, Bristol, United Kingdom-100 μM) was added to the aCSF in order to block GABA_A_ receptors. All drugs [CP 55,940 (Tocris BioScience, Bristol, United Kingdom), WIN 55.212-2 (Win-2)(Tocris BioScience, Bristol, United Kingdom), AM251 (Tocris BioScience, Bristol, United Kingdom), MPEP (Tocris BioScience, Bristol, United Kingdom), CPCCoEt (Tocris BioScience, Bristol, United Kingdom), THL (Santa Cruz Biotechnology, CA, EEUU), RHC-80267 (Tocris BioScience, Bristol, United Kingdom), URB597 (Tocris BioScience, Bristol, United Kingdom), AMG9810 (Tocris BioScience, Bristol, United Kingdom), D-APV (Tocris BioScience, Bristol, United Kingdom)] were dissolved in dimethyl sulfoxide (DMSO; Sigma-Aldrich, Madrid, Spain) and added in their final concentrations to aCSF. The stimulation electrode was placed in the MPP and the recording pipette in the inner one-third of the DGML (Figure 1F). A low-frequency stimulation (LFS, 10 min at 10 Hz) was applied to induce eCB-eLTD at glutamatergic inputs following recordings of a steady baseline in the presence of drugs. The fEPSP area was measured. The magnitude of the CB1-eLTD after LFS was calculated as the percentage between baseline (averaged excitatory responses for 10 min before LFS) and the last 10 min of stable responses 30 min after LFS [17].

### 2.6. Statistical Analysis

Before analysis, Shapiro-Wilk and Kolmogorov–Smirnov tests were used for examining normal distribution of the data. Homogeneity of variances was determined by Levene’s test. Two-way ANOVA analysis was performed to evaluate DID and EE effects as well as the interaction between both conditions. To further explore the effect of DID and EE in different experimental conditions (sham, EtOH, EE-EtOH, and EE-H_2_0), one-way ANOVA with post hoc analysis (Bonferroni correction for equal variances or Tamhane’s T2 correction for unequal variances) was used. Data obtained from the Barnes maze test were analyzed using two-way ANOVA’s multiple comparison. BEC differences between the control and EtOH groups and statistical significance data (EE-EtOH vs. EE-EtOH + drugs and EE-H_2_0 vs. EE-H_2_0 + drugs) were analyzed using Student’s *t*-test. *p* ≤ 0.05 was considered statistically significant. All statistical tests were performed with SPSS statistical software (version 22.0, IBM, Barcelona, Spain) and data were given as mean ± standard error of the mean (SEM) with *p*-values and sample size (*n*).

## 3. Results

### 3.1. EE Recovers EtOH-Induced Memory Impairment

Young adult mice exposed to EtOH during adolescence showed significant higher latency to escape (Figure 2A, two-way ANOVA’s multiple comparison (*n* = 12); day 1 ** *p* = 0.004, day 2 * *p* = 0.021, day 3 * *p* = 0.033, and day 5 * *p* = 0.034) and more errors committed in the Barnes maze than controls (Figure 2B, two-way ANOVA’s multiple comparison; day 1 * *p* = 0.027). These results suggest a direct negative effect of adolescent BD on memory in young adults. EE in early adulthood was able to rescue the memory impairment induced by EtOH. Thus, a lower latency to escape (Figure 2A, two-way ANOVA’s multiple comparison (*n* = 12); day 1 ^+^ *p* = 0.014, day 5 ^+^ *p* = 0.046) and fewer errors (Figure 2B, two-way ANOVA’s multiple comparison (*n* = 12) day 1 ^+^ *p* = 0.032, day 2 ^+^ *p* = 0.025, day 3 ^+^ *p* = 0.043 and day 4 ^+^ *p* = 0.028) were observed after EE. Furthermore, the two-way ANOVA revealed the significant effect of DID (F_(1.38)_ = 12.42, *** *p* = 0.001), EE (F_(1.38)_ = 10,80, ** *p* = 0.002), and their interaction on latency (F_(1.38)_ = 24,69, *** *p* = 0.000), as well as on errors (DID: F_(1.39)_ = 6.32, * *p* = 0.016; EE: F_(1.39)_ = 14,22, *** *p* = 0.001), and their interaction (F_(1.39)_ = 11.80, *** *p* = 0.001).

EtOH also had a negative impact on the process in which the mice solved the maze. Thus, EtOH-exposed mice showed mostly random strategies (Figure 2C, one-way ANOVA; *** *p* = 0.000 vs. sham), with a reduction in serial, and, particularly, spatial strategies (Figure 2D,E). However, control mice solved the maze mainly using a serial strategy (Figure 2D, one-way ANOVA (*n* = 12); ** *p* = 0.0094 vs. EtOH). The EE was able to significantly revert the changes in random (Figure 2C, one-way ANOVA (*n* = 12); ^+++^ *p* = 0.001) and spatial strategies induced by EtOH (Figure 2E, one-way ANOVA (*n* = 12); ^+++^ *p* = 0.000). Altogether, the EE applied in early adulthood recovered the persistent memory impairment observed after BD.

### 3.2. EE Recovers the CB1 Receptor-Mediated Excitatory Synaptic Transmission and MPP-LTD Lost after EtOH

**Figure 3:**
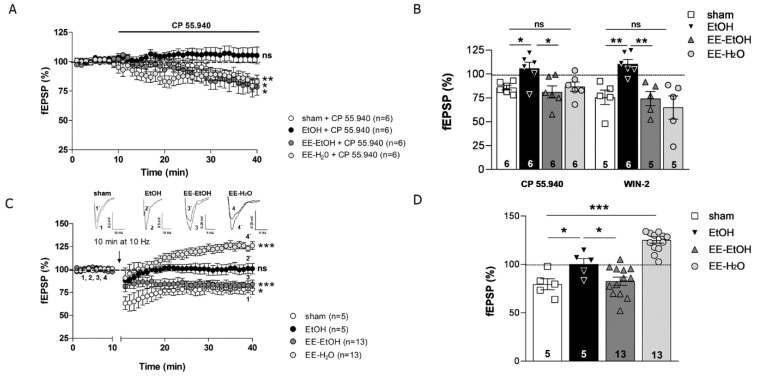
EE recovers CB1R-mediated excitatory transmission and MPP-LTD impaired by EtOH intake during adolescence. EE in young adult brain leads to MPP-LTP. (**A**) CP 55,940 (10 μM, white circles) reduces fEPSPs in sham but not in EtOH-treated mice (black circles). EE recovers the fEPSPs impaired by EtOH intake during adolescence (dark grey circles). CP 55,940 (10 μM, light grey circles) depresses fEPSPs in EE-H_2_0. Data are expressed as mean ± SEM. Student’s *t*-test, *p* > 0.05 (ns = not significant); * *p* ≤ 0.05; ** *p* ≤ 0.01 vs. baseline. (**B**) Summary bars: sham + CP 55,940 (10 μM), EtOH + CP 55,940 (10 μM), EE-EtOH + CP 55,940 (10 μM), EE-H_2_O + CP 55,940 (10 μM); sham + Win-2 (5 μM), EtOH + Win-2 (5 μM), EE-EtOH + Win-2 (5 μM), EE-H_2_O + Win-2 (5 μM). Numbers in the bars are individual experiments. Data are expressed as mean ± SEM. One-way ANOVA, *p* > 0.05 (ns = not significant); * *p* ≤ 0.05; ** *p* ≤ 0.01. (**C**) Up; superimposed traces (average of 10 consecutive fEPSPs taken at the time indicated on the time course) showing the effect after LFS (10 min, 10 Hz). Down; LFS triggers MPP-LTD in sham (white circles) but not in EtOH (black circles). EE-EtOH rescues MPP-LTD (dark grey circles) and LFS leads to MPP-LTP in EE-H_2_0 (light grey circles). Data are expressed as mean ± SEM. Student’s *t*-test, *p* > 0.05 (ns = not significant); * *p* ≤ 0.05; *** *p* ≤ 0.001 vs. baseline. (**D**) Summary bar graph of MPP-LTD and MPP-LTP experiments: sham, EtOH, EE-EtOH, and EE-H_2_0. Numbers in the bars are individual experiments. Data are expressed as mean ± SEM. One-way ANOVA, * *p* ≤ 0.05; *** *p* ≤ 0.001.

The CB1 receptor-dependent depression of the excitatory synaptic transmission at the MPP-GC synapses (Figure 3A, Student’s *t*-test (*n* = 6); CP 55,940, 10 μM sham: 85.76 ± 2.37% of fEPSP; ** *p* = 0.002 vs. baseline) was absent in young adult mice after adolescent EtOH intake (Figure 3A, Student’s *t*-test (*n* = 6); CP 55,940, 10 μM EtOH: 105.95 ± 6.18% of fEPSP; *p* = 0.473 vs. baseline). Direct activation of CB1R in the DG by CP 55,940 or WIN-2 showed differences between standard rearing control and EtOH (Figure 3B, one-way ANOVA; CP 55,940, 10 μM, * *p* = 0.049 and WIN-2, 5 μM, ** *p* = 0.006), as we previously described [17,18]. However, CP 55,940 (Figure 3A, Student’s *t*-test (*n* = 6); CP 55,940, 10 μM EE-EtOH: 81.30 ± 6.28% of fEPSP; * *p* = 0.03 vs. baseline) and WIN-2 were able to induce synaptic depression in EtOH mice after EE during withdrawal (Figure 3B, one-way ANOVA (*n* = 6); CP 55,940, 10 μM, * *p* = 0.014 vs. EtOH and WIN-2, 5 μM (*n* = 5), ** *p* = 0.004 vs. EtOH). Two-way ANOVA analysis revealed a significant effect of DID (F_(1.13)_ = 15.09, *** *p* = 0.001), EE (F_(1.13)_ = 16.26, ** *p* = 0.002), and the interaction between them on MPP synaptic transmission (F_(1.13)_ = 430.09, *** *p* = 0.000). Furthermore, the LTD elicited at the MPP-GC synapses by MPP fiber stimulation (10 Hz for 10 min) under normal conditions [18] (Figure 3C, Student’s *t*-test (*n* = 5) 79.13 ± 5.96% of fEPSP; * *p* = 0.024 vs. baseline) that was absent after adolescent EtOH [17] (Figure 3C, Student’s *t*-test (*n* = 5) 100.36 ± 5.5% of fEPSP; *p* = 0.94 vs. baseline) appeared to be rescued by EE in young adults (Figure 3C, Student’s *t*-test (*n* = 13) 82.50 ± 4.24% of fEPSP; *** *p* = 0.000 vs. baseline). Both the LTD loss in EtOH and its rescue after EE were statistically significant (Figure 3D, one-way ANOVA (*n* = 5) * *p* = 0.0035 EtOH vs. sham; (*n* = 13) * *p* = 0.003 EtOH vs. EE-EtOH). Again, a significant effect of DID (F_(1.20)_ = 5.41, * *p* = 0.031), EE (F_(1.20)_ = 5.68, * *p* = 0.027) and the interaction between DID and EE was observed on MPP-LTD (F_(1.20)_ = 678.88, *** *p* = 0.000). Altogether, these findings demonstrate that EE can overcome the deficits in CB1R-mediated excitatory synaptic transmission and MPP-LTD detected in the mature brain after EtOH intake during adolescence.

### 3.3. EE Promotes MPP-LTP in Young Adult Mice

As in sham [18], CP 55,940 (10 μM) decreased the excitatory synaptic transmission at MPP-GC synapses in EE mice exposed to water only (EE-H_2_0) [Figure 3A, CP 55,940, 10 μM (*n* = 6) 86.70 ± 4.90% of fEPSP; Student’s *t*-test, * *p* = 0.042 vs. baseline]. Moreover, LFS of MPP triggered an overt MPP-LTP in EE-H_2_0 mice [Figure 3C Student’s *t*-test (*n* = 13); 124.50 ± 3.54% of fEPSP; *** *p* = 0.000 vs. baseline]. This MPP-LTP was remarkably significant [Figure 3D one-way ANOVA; *** *p* = 0.000 vs. sham].

### 3.4. EE Involves CB1R, Group I mGluRs and 2-AG in MPP-LTD Recovery after Adolescent EtOH

**Figure 4:**
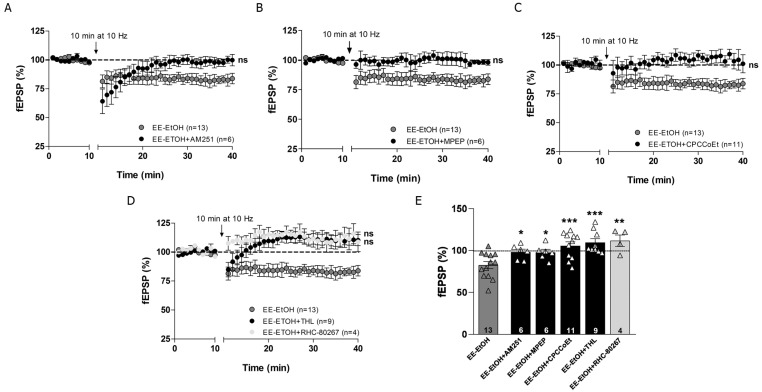
Activation of CB1R, mGluRs and 2-AG are required for MPP-LTD in EE-EtOH. (**A**) AM251 (4 μM, black circles; Student’s *t*-test; *p* > 0.05 vs. baseline; ns = not significant), (**B**) MPEP (10 μM; black circles; Student’s *t*-test; *p* > 0.05 vs. baseline; ns = not significant), and (**C**) CPCCoEt (50 μM; black circles; Student’s *t*-test; *p* > 0.05 vs. baseline; ns = not significant), block MPP-LTD in EE-EtOH (dark grey circles). (**D**) MPP-LTD (dark grey circles) in EE-EtOH is abolished by THL (10 μM; dark circles; Student’s *t*-test; *p* > 0.05 vs. baseline; ns = not significant) and RHC-80267 (100 μM; light grey circles: Student’s *t*-test; *p* > 0.05 vs. baseline, respectively; ns = not significant). (**E**) Summary bars: EE-EtOH + AM251 (*n* = 6) (4 μM) EE-EtOH + MPEP (*n* = 6) (10 μM), EE-EtOH + CPCCoEt (*n* = 11) (50 μM), EE-EtOH + THL (*n* = 9) (10 μM), EE-EtOH + RHC-80267 (*n* = 4) (100 μM). Student’s *t*-test; * *p* ≤ 0.05; ** *p* ≤ 0.01; *** *p* ≤ 0.001 vs. EE-EtOH. Numbers in the bars are individual experiments. Data expressed as mean ± S.E.M.

The MPP-LTD rescued by EE was blocked by the CB1R inverse agonist AM251 (4 µM) (Figure 4A,E, Student’s *t*-test (*n* = 6) 98.07 ± 3.51% of fEPSP; *p* = 0.589 vs. baseline; * *p* = 0.013 vs. EE-EtOH). This CB1 receptor-dependent eLTD was also blocked by the mGluR5 antagonist MPEP (10 μM) (Figure 4B,E, Student’s *t*-test (*n* = 6) 97.82 ± 3.64% of fEPSP; *p* = 0.531 vs. baseline; * *p* = 0.015 vs. EE-EtOH) and the mGluR1 antagonist CPCCoEt (50 μM) (Figure 4C,E, Student’s *t*-test (*n* = 11) 105.63 ± 4.67% of fEPSP; *p* = 0.395 vs. baseline; *** *p* = 0.001 vs. EE-EtOH), indicating that both group I mGluRs were involved in MPP-LTD after EE in EtOH mice. Furthermore, the diacylglycerol lipase inhibitors tetrahydrolipstatin (THL, 10 μM) (Figure 4D,E, Student’s *t*-test (*n* = 9) 109.45 ± 4.53% of fEPSP; *p* = 0.073 vs. baseline; *** *p* = 0.000 vs. EE-EtOH) and RHC-80267 (100 μM) (Figure 4D,E Student’s *t*-test (*n* = 4) 111.17 ± 6.94% of fEPSP; *p* = 0.188 vs. baseline; ** *p* = 0.004 vs. EE-EtOH) completely abolished MPP-LTD in EE-EtOH mice, indicating that 2-AG synthesis was also required. Altogether, these data suggest that EE influences cannabinoid-dependent synaptic plasticity to counteract the impaired MPP-LTD in young adults exposed to high EtOH consumption during adolescence [17].

### 3.5. MPP-LTP Elicited by EE Requires TRPV1 and AEA

**Figure 5:**
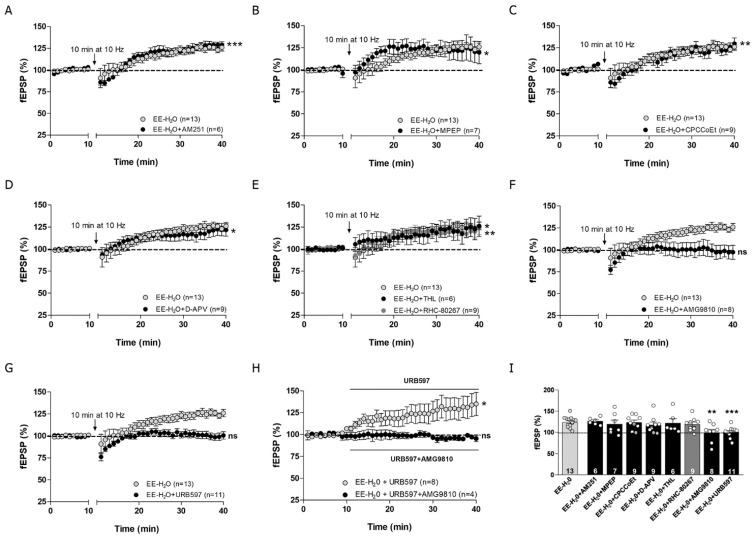
Activation of TRPV1 and AEA are required for MPP-LTP in EE-H_2_O. (**A**) AM251 (4 μM, black circles; Student’s *t*-test; *** *p* ≤ 0.001 vs. baseline), (**B**) MPEP (10 μM; black circles; Student’s *t*-test; * *p* ≤ 0.05 vs. baseline), (**C**) CPCCoEt (50 μM; black circles; Student’s *t*-test; ** *p* ≤ 0.01 vs. baseline), (**D**) D-APV (50 μM; black circles; Student’s *t*-test; * *p* ≤ 0.05 vs. baseline) and (**E**) THL (10 μM; black circles; Student’s *t*-test * *p* ≤ 0.05 vs. baseline) and RHC-80267 (100 μM; dark grey circles: Student’s *t*-test; ** *p* ≤ 0.01 vs. baseline) do not affect MPP-LTP in EE-H_2_O mice. (**F**) However, MPP-LTP is blocked by AMG9810 (3 μM; black circles; Student’s *t*-test; *p* > 0.05 vs. baseline; ns = not significant) and (**G**) inhibited by URB597 (2 μM, >20 min; black circles; Student’s *t*-test; *p* > 0.05 vs. baseline; ns = not significant). (**H**) The increase in fEPSP by URB597 (2 μM, >20 min; grey circles; Student’s *t*-test; * *p* ≤ 0.05 vs. baseline) is abolished by AMG9810 (3 μM; black circles; Student’s *t*-test; *p* > 0.05 vs. baseline; ns = not significant). (**I**) Summary bar histogram: EE-H_2_O + (*n* = 6) AM251 (4 μM), EE-H_2_O + MPEP (*n* = 7) (10 μM), EE-H_2_O + CPCCoEt (*n* = 9) (50 μM), EE-H_2_O + D-APV (*n* = 9) (50 μM), EE-H_2_O + THL (*n* = 6) (10 μM), EE-H_2_O + RHC-80267 (*n* = 9) (100 μM), EE-H_2_O + AMG9810 (*n* = 8) (3 μM), and EE-H_2_O + URB597 (*n* = 11) (2 μM) Student’s *t*-test; *p* > 0.05; ** *p* ≤ 0.01; *** *p* ≤ 0.001 vs. EE-H_2_O. Numbers in the bars are individual experiments. Data expressed as mean ± S.E.M.

The LTP observed at the MPP-GC synapses in EE mice only exposed to water was unaffected by AM251 (4 μM) (Figure 5A,I, Student’s *t*-test (*n* = 6) 126.23 ± 3.41% of fEPSP; *** *p* = 0.001 vs. baseline; *p* = 0.715 vs. EE-H_2_0), MPEP (10 μM) (Figure 5B,I, (*n* = 7) Student’s *t*-test 123.81 ± 1.79% of fEPSP; * *p* = 0.05 vs. baseline; *p* = 0.546 vs. EE-H_2_0), CPCCoEt (50 μM) (Figure 5C,I, Student’s *t*-test (*n* = 9) 123.36 ± 5.52% of fEPSP; ** *p* = 0.003 vs. baseline; *p* = 0.862 vs. EE-H_2_0), D-APV (50 μM) (Figure 5D,I, Student’s *t*-test (*n* = 9) 117.10 ± 6.61% of fEPSP; * *p* = 0.028 vs. baseline; *p* = 0.378 vs. EE-H_2_0), THL (10 μM) (Figure 5E,I, (*n* = 6) Student’s *t*-test 123.48 ± 8.56% of fEPSP; * *p* = 0.034 vs. baseline; *p* = 0.810 vs. EE-H_2_0), and RHC-80267 (100 μM) (Figure 5E,I, Student’s *t*-test (*n* = 9) 118.70 ± 5.27% of fEPSP; ** *p* = 0.008 vs. baseline; *p* = 0.394 vs. EE-H_2_0). Altogether, these data indicate that CB1Rs, mGluR1, mGluR5, NMDAR, and 2-AG were not involved in this form of LTP. However, MPP-LTP was blocked by the TRPV1 receptor antagonist AMG9810 (10 μM) (Figure 5F,I, Student’s *t*-test (*n* = 8) 99.37 ± 7.71% of fEPSP; *p* = 0.937 vs. baseline; ** *p* = 0.003 vs. EE-H_2_0) and the fatty acid amide hydrolase (FAAH) inhibitor URB597 (2 μM) (Figure 5G,I, Student’s *t*-test (*n* = 11) 100.80 ± 3.88% of fEPSP; *p* = 0.796 vs. baseline; *** *p* = 0.000 vs. EE-H_2_0), indicating that TRPV1 and AEA are necessary to induce MPP-LTP in mice exposed to EE. Furthermore, MPP synaptic transmission was increased in the presence of URB597 (Figure 5H, Student’s *t*-test (*n* = 8) 130.47 ± 12.45% of fEPSP; * *p* = 0.044 vs. baseline) and abolished after URB597 and AMG9810 co-application (Figure 5H, Student’s *t*-test (*n* = 4) 95.99 ± 2.46% of fEPSP; *p* = 0.193 vs. baseline).

## 4. Discussion

Although EE has been demonstrated to rescue memory impairment [5,34], the role of EE in LTD is not well understood [21]. We have shown in this study that young adult male mice exposed to EE recover the 9group I mGluR-dependent MPP-LTD that is lost after adolescent EtOH consumption [17,18].

### 4.1. Mechanisms of MPP-LTD Rescue by EE in Adult Mice after Adolescent EtOH Intake

The EE rescue of MPP-LTD involves several components of the canonical endocannabinoid signaling pathway: CB1R, group I mGluRs and 2-AG [18,35]. Noticeably, the mice had MPP-LTD deficits although the amount of EtOH intake was modest compared with the BEC criteria (≥0.08 g/dL) set for a pattern of adolescent BD [36]. Moderate EtOH intake during the critical adolescent period of brain development is sufficient to promote long lasting ECS-dependent changes in hippocampal plasticity [17] and cognition [5]. Both the ECS and EtOH are under mutual influence, as the former modulates EtOH-motivated behavior, and the latter profoundly dysregulates the ECS [37]. Thereby, the long-lasting harmful changes in hippocampal mGluR5 mRNA, CB1 receptor expression, localization, and function at MPP synapses caused by adolescent EtOH intake associate with recognition memory deficits that can be recovered with rising 2-AG [17,18,32].

The exposure to EE protects against neuronal and cognitive damage, reduces EtOH-induced reinforcing, and recovers different memory forms and motor coordination in adult mice exposed to adolescent EtOH intake [5,17,38]. EE stimulates hippocampal neurogenesis [39] and increases nerve growth factor and brain-derived neurotrophic factor (BDNF) [40,41]. These processes elicit neuroprotective responses and induce neural plasticity and synaptic structural brain changes [42] leading to an improvement of spatial navigation [43], learning, and spatial memory [5]. Furthermore, many synaptic proteins and ionotropic glutamate receptors involved in plasticity and memory can be changed by EE [34,44]. Among them, mGluR5, which participates in the EE-mediated BDNF increase [45], rises during EE conditions [46]. In addition, EE increases glutamate release [47] and decreases the neuronal glutamate transporter EAAC1 [48]. These combined events favor a greater extracellular glutamate milieu, eventually leading to group I mGluRs activation and mGluR-dependent synaptic plasticity. mGluR1 antagonism impairs LTD [49] and an mGluR5 blockade prevents LTD that is necessary for spatial learning and memory [50]. However, EtOH elicits a significant reduction in CB1R expression and receptor labeling, particularly in the middle one-third of the DGML targeted by MPP synaptic terminals [15,17]. CB1 receptor signaling is also affected by EtOH intake as a consequence of reduced CB1 receptor binding and decreased Gαi2 subunit expression, which is known to be linked to learning and social behavior [14,17]. Although 2-AG and DAGL expression does not seem to change, a drastic increase in arachidonic acid and MAGL was noticed in a similar EtOH intake model used in this study [17]. Therefore, 2-AG levels are likely to increase in our model [51], eventually balanced by more 2-AG degradation through MAGL increase [52]. DAGL inhibitors abolished the EE-induced MPP-LTD recovery, indicating that 2-AG is involved. Conversely, the enhancement of long-term synaptic plasticity and cognitive performance by MAGL suppression implies CB1Rs [53]. Our previous observations showed that MAGL inhibition rescues MPP-LTD and recognition memory in adult mice after EtOH treatment during adolescence [17]. These observations suggest that the 2-AG increase surmounts the CB1R reduction at MPP synapses, potentially due to the high coupling efficiency of CB1 receptors in glutamatergic terminals [54]. Thus, under EE conditions, the plausible increase in glutamate levels favored by EE after LFS will activate the available mGluR5 (and potentially mGluR1), leading to the rise in 2-AG synthesis and presynaptic CB1R activation. The net effect of EE on endocannabinoid levels and the expression of ECS components needs to be determined.

### 4.2. EE Prompts MPP-LTP in Water-Exposed Mice

Although CB1R activation reduced fEPSPs after EE in the mice who drank water to the same extent as the controls, they also exhibited MPP-LTP, which is triggered by the same LFS that elicits MPP-LTD [18]. This LTP involves TRPV1 and AEA, but not CB1Rs, group I mGluRs, or 2-AG. The potentiation of the MPP synaptic strength aligns with the transient potentiation of the perforant path synapses after EE [55]. In this sense, spatial learning associates with hippocampal LTP and EE improves both LTP and spatial learning [45]. EE also modifies the expression of presynaptic and postsynaptic proteins, including AMPA and NMDA receptor subunits, as well as LTP-related molecules such as Ca2+/calmodulin-dependent Kinase II and cAMP response element binding protein [34]. However, the observed MPP-LTP following LFS after EE did not seem to depend on NMDA receptors, as D-APV had no effect. Previous studies have shown that voluntary exercise promotes MPP-LTP [56] and, similar to EE, augments BDNF [57] that favors neurotransmitter release and LTP [34]. Group I mGluRs are also involved in hippocampal LTP [21]. In our study, neither mGluR1 nor mGluR5 seemed to participate in the EE-elicited MPP-LTP after LFS, as LTP was not changed by the antagonism of group I mGluRs. However, we cannot rule out that other MPP stimulation patterns may activate group I mGluRs, since the opposed effects of EE on LTP are described [58]. Furthermore, bidirectional LTD/LTP allows a switch at a given synapse, enabling adaptive changes in synaptic strength, and controlled by the level and timing of endocannabinoids [59]. The MPP-LTP observed upon EE in water conditions required AEA, as the FAAH inhibitor URB597 occluded the potentiation. This suggests that AEA regulation may be a limiting factor for MPP-LTP. AEA is an endogenous TRPV1 agonist and is restricted to certain brain cells and regions [60], facilitates glutamate release at excitatory synapses, and mediates LTD in the CA1 hippocampus [61]. TRPV1 in granule cell dendritic spines intervenes (together with mGluR5, postsynaptic calcium, and AEA) in LTD at MPP synapses [62]. Recent evidence indicates that the lack of TRPV1 alters the main 2-AG and AEA degrading enzymes, as well as CB1Rs localized to excitatory and inhibitory terminals in the outer 2/3 DGML. Thereby, a crosstalk between TRPV1 and CB1R has been proposed [63]. In this scenario, our findings support a switch from MPP-LTD to MPP-LTP when EE is applied. Our findings also suggest that EE modifies the intensity threshold of excitatory synapses. We observed that the switch in long-term plasticity was not dependent on CB1Rs, since the MPP-LTP was not blocked by AM251. Endocannabinoids can also trigger LTP in the hippocampus through stimulation of CB1Rs in astrocytes. However, LTP requires group I mGluRs activated by the release of astrocytic glutamate at distant synapses [64]. This mechanism seems to be unlikely in our model as MPP-LTP was not dependent on group I mGluRs.

In the case of excitatory synapses, postsynaptic calcium has been proposed as a principal mediator for bidirectional LTP/LTD [65]. In this sense, the MPP-LTP after EE may be triggered by an increase in glutamate release and intracellular calcium that can stimulate AEA production. Moreover, PLC activation by a rise in calcium generates 2-AG [66], which may yield to 1-AG due to acyl migration. This may eventually lead to TRPV1 activation [67]. In addition, PLC activity seems to be critical for TRPV1 since the receptor is sensitized by PLC-mediated hydrolysis of phosphoinositides (PI) and its activity is augmented by PI decrease [68].

Intracellular TRPV1 may regulate calcium release from the sarco/endoplasmic reticulum needed for excitatory synaptic plasticity [69]. The biosynthetic AEA enzyme, N-acyl phosphatidylethanolamine phospholipase D (NAPE-PLD), is highly expressed in dentate granule cells [70]. NAPE-PLD is found in the smooth endoplasmic reticulum of excitatory presynaptic terminals [71], as well as in postsynaptic dendrites and spines receiving excitatory synapses [72]. Hence, the generation of AEA or other N-acylethanolamines by the calcium-dependent catalytic activity of NAPE-PLD may cause postsynaptic TRPV1 activation.

Altogether, the cognitive improvement elicited by EE was similar in adult mice exposed to water and those exposed to EtOH during adolescence [8]. Thus, MPP-LTP elicited by EE may be implicated in this ceiling effect in which TRPV1 and AEA seem to be critical players.

## Data Availability

Not applicable.
